# Supplementary material for: Ezrin, radixin, and moesin are dispensable for macrophage migration and cellular cortex mechanics
Source: EMBO J. 2024 Jul 18;43(21):4. doi: 10.1038/s44318-024-00173-7 (PMC11535515; doi:10.1038/s44318-024-00173-7)
Supplement: Supplementary file 1 — Appendix [file 44318_2024_173_MOESM1_ESM.pdf]

Appendix

Table of contents

Appendix Fig S1: ERM-tKO clones have genomic mutations on each 3 ERM genes………….2

Appendix Fig S2: Quantification of podosome stability on RIM videos.………………………….3

Verdys et al. Appendix Fig. S1

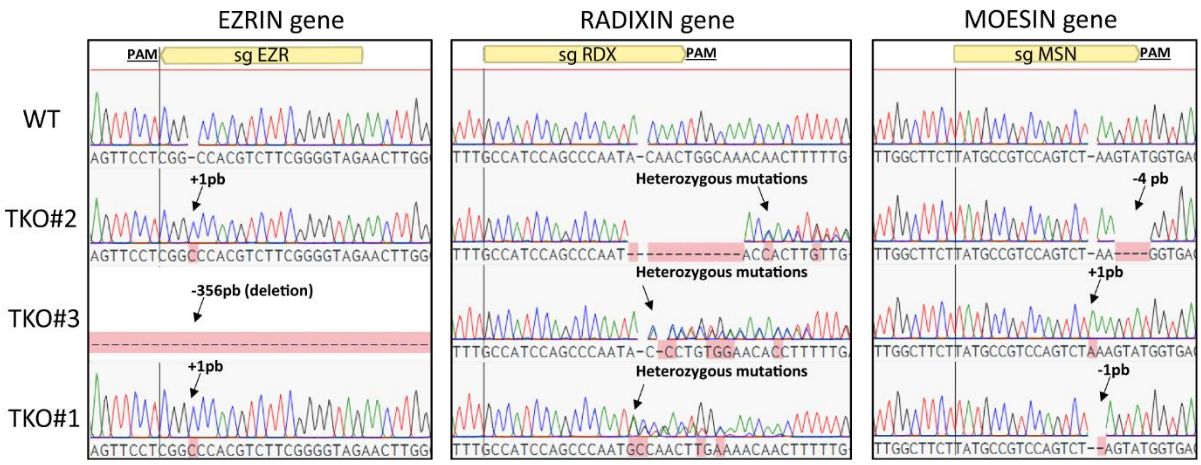

Appendix Fig S1: ERM-tKO clones have genomic mutations on each 3 ERM genes

Sequence analysis of genomic DNA mutations of the three ERM-tKO clones (tKO#1, tKO#2, and tKO#3) in ezrin, radixin and moesin genes. Targeted sgRNAs and PAM are depicted.

Verdys et al. Appendix Fig S2

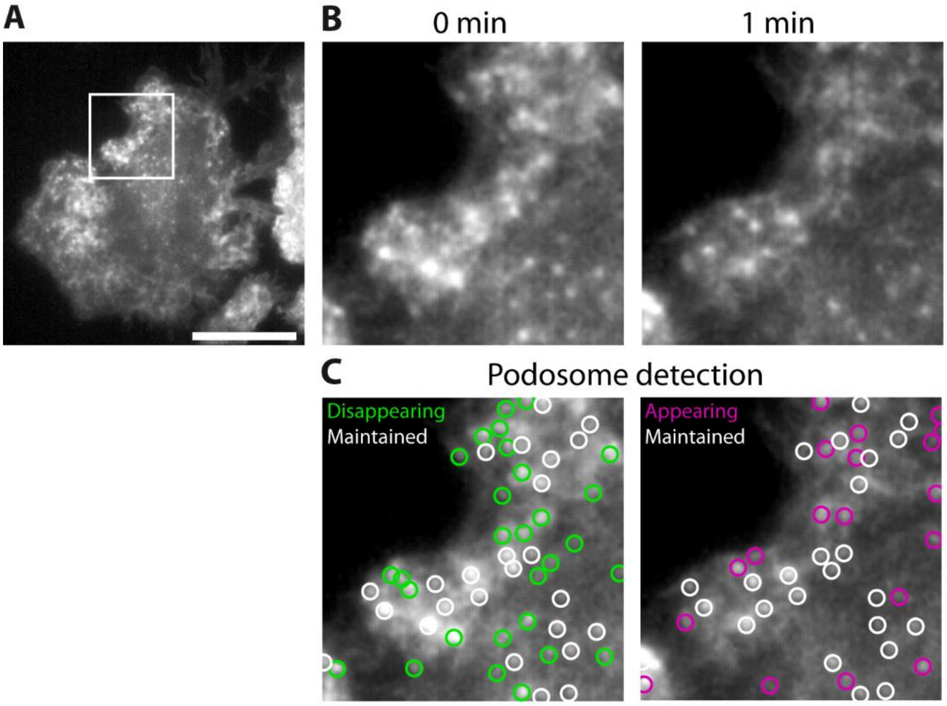

Appendix Fig S2: Quantification of podosome stability on RIM videos.

We designed an in-house imageJ macro to extract podosome stability from RIM videos of WT and ERM-tKO macrophages expressing lifeact-GFP **(A)**. Briefly, one image per minute was selected for the first 3 minutes of the video (B), then podosome location and shape was extracted and a “Maxima Within Tolerance” mask was generated. The comparison between consecutive masks allowed podosome classification into three categories (Appearing, disappearing and maintained) depending in their presence at first, second or both images (C).
