# Supplementary figures and images for: Ezrin, radixin, and moesin are dispensable for macrophage migration and cellular cortex mechanics
Source: EMBO J. 2024 Jul 18;43(21):4. doi: 10.1038/s44318-024-00173-7 (PMC11535515; doi:10.1038/s44318-024-00173-7)

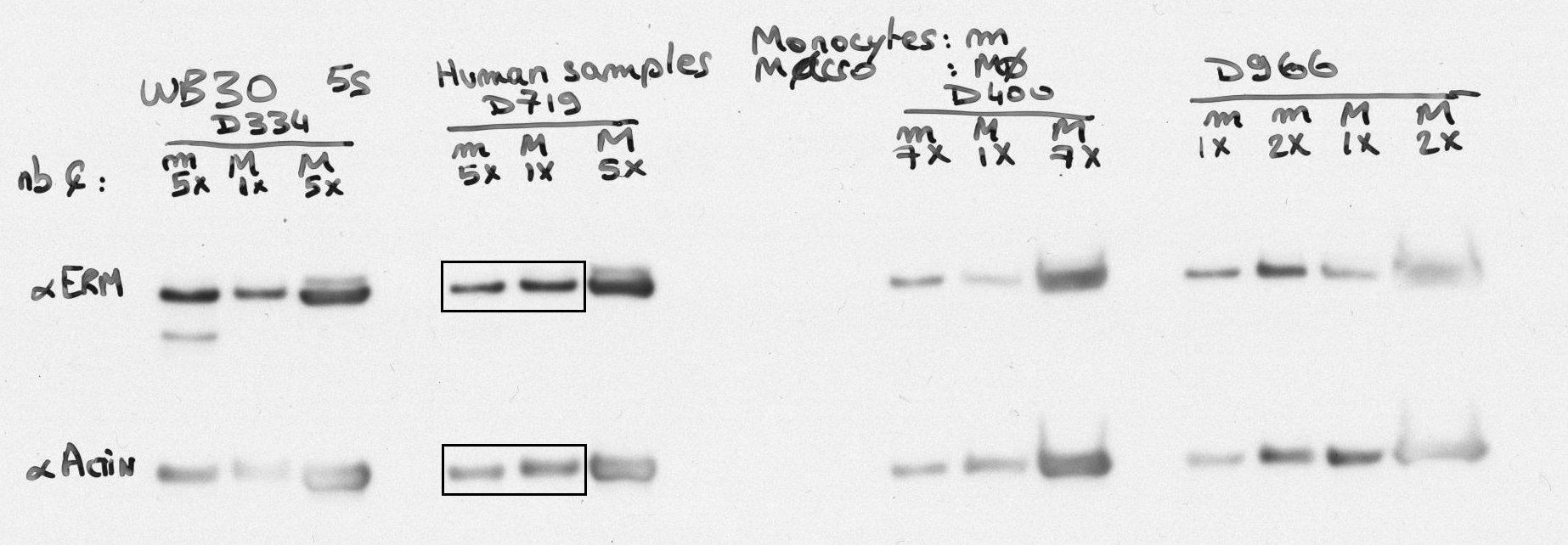

Supplement: Supplementary file 19 — Source data Fig. 1 [file 44318_2024_173_MOESM19_ESM.zip › Figure 1_ human macro_siMSN_Hox KO simples/1A Image Blot_human ERM/1A_ERM_Actin_WB30-5s.tif]

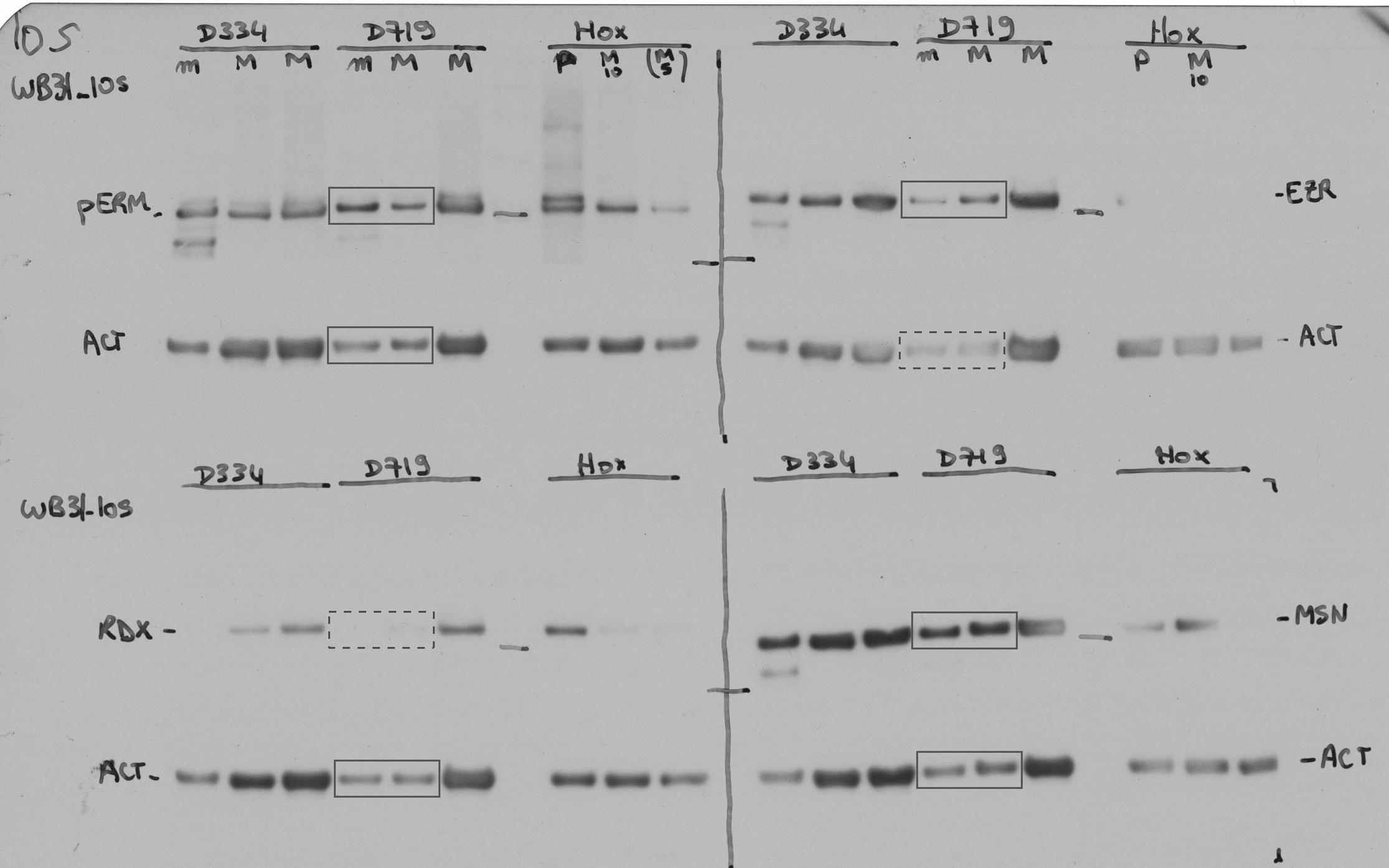

Supplement: Supplementary file 19 — Source data Fig. 1 [file 44318_2024_173_MOESM19_ESM.zip › Figure 1_ human macro_siMSN_Hox KO simples/1A Image Blot_human ERM/1A_Ezr_Msn_pERM_WB31_10s.tif]

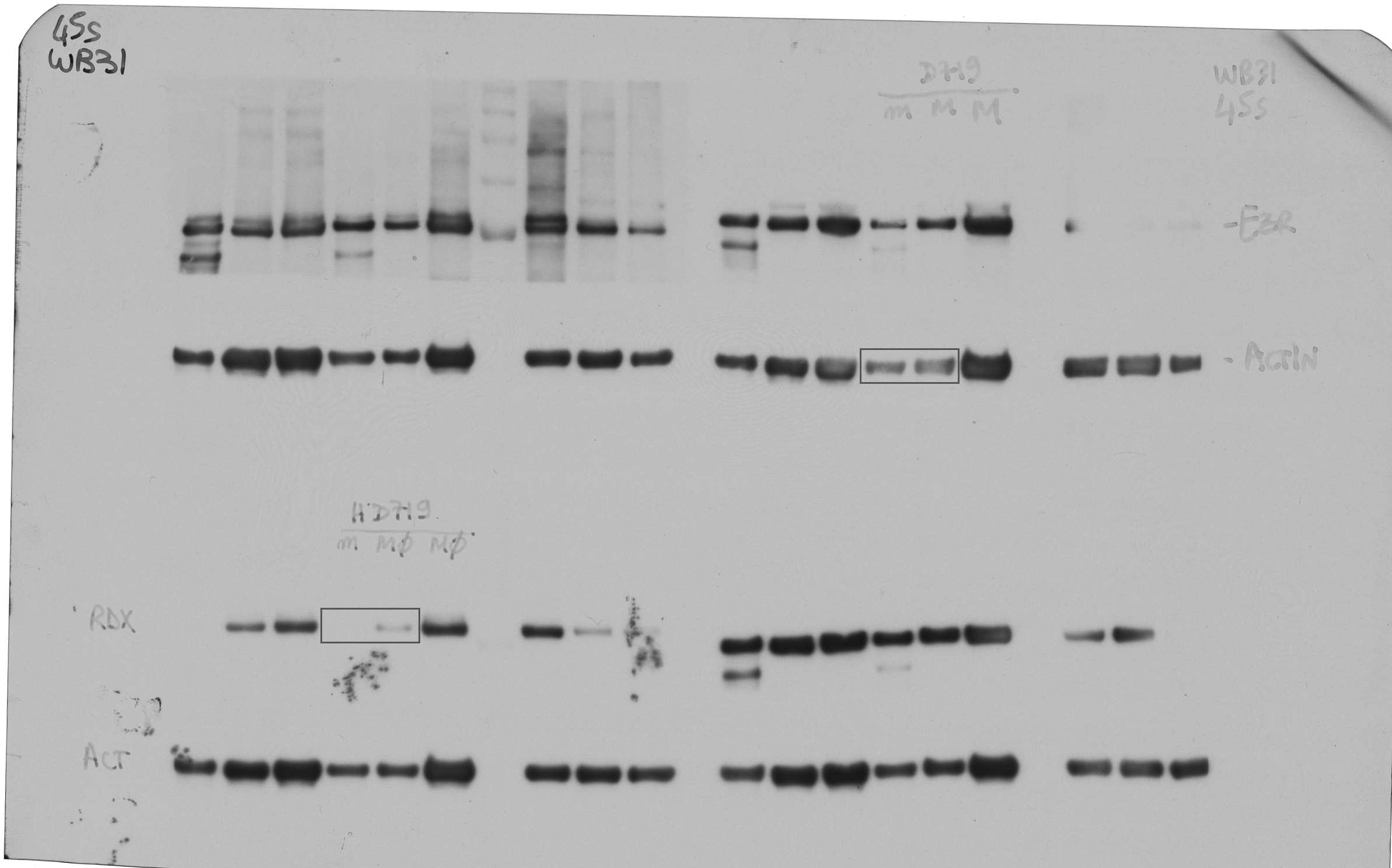

Supplement: Supplementary file 19 — Source data Fig. 1 [file 44318_2024_173_MOESM19_ESM.zip › Figure 1_ human macro_siMSN_Hox KO simples/1A Image Blot_human ERM/1A_Rdx_Actin of Ezrin_WB31_45s.tif]

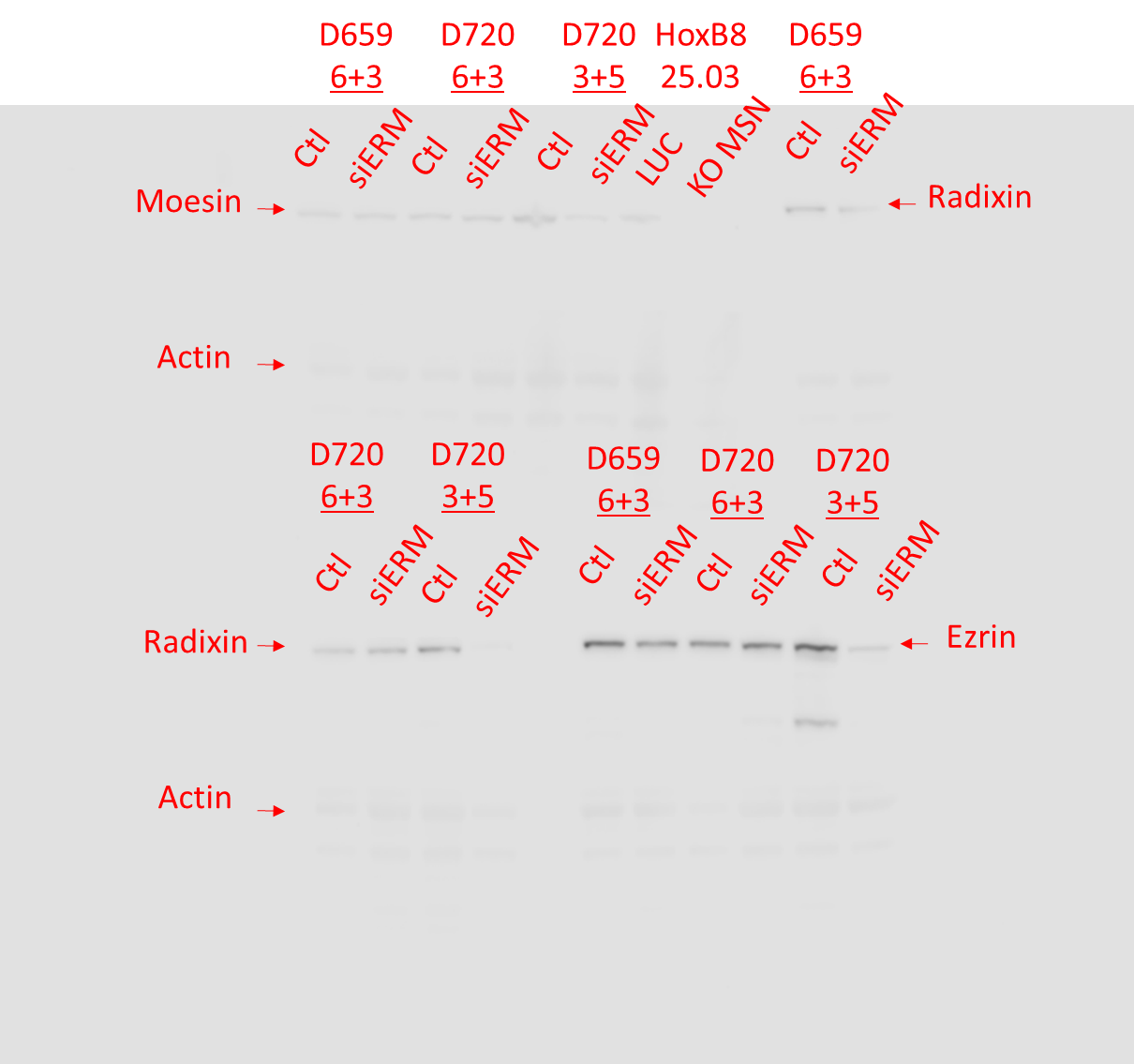

Supplement: Supplementary file 19 — Source data Fig. 1 [file 44318_2024_173_MOESM19_ESM.zip › Figure 1_ human macro_siMSN_Hox KO simples/1C WB/D720 original annotated low contrast.png]

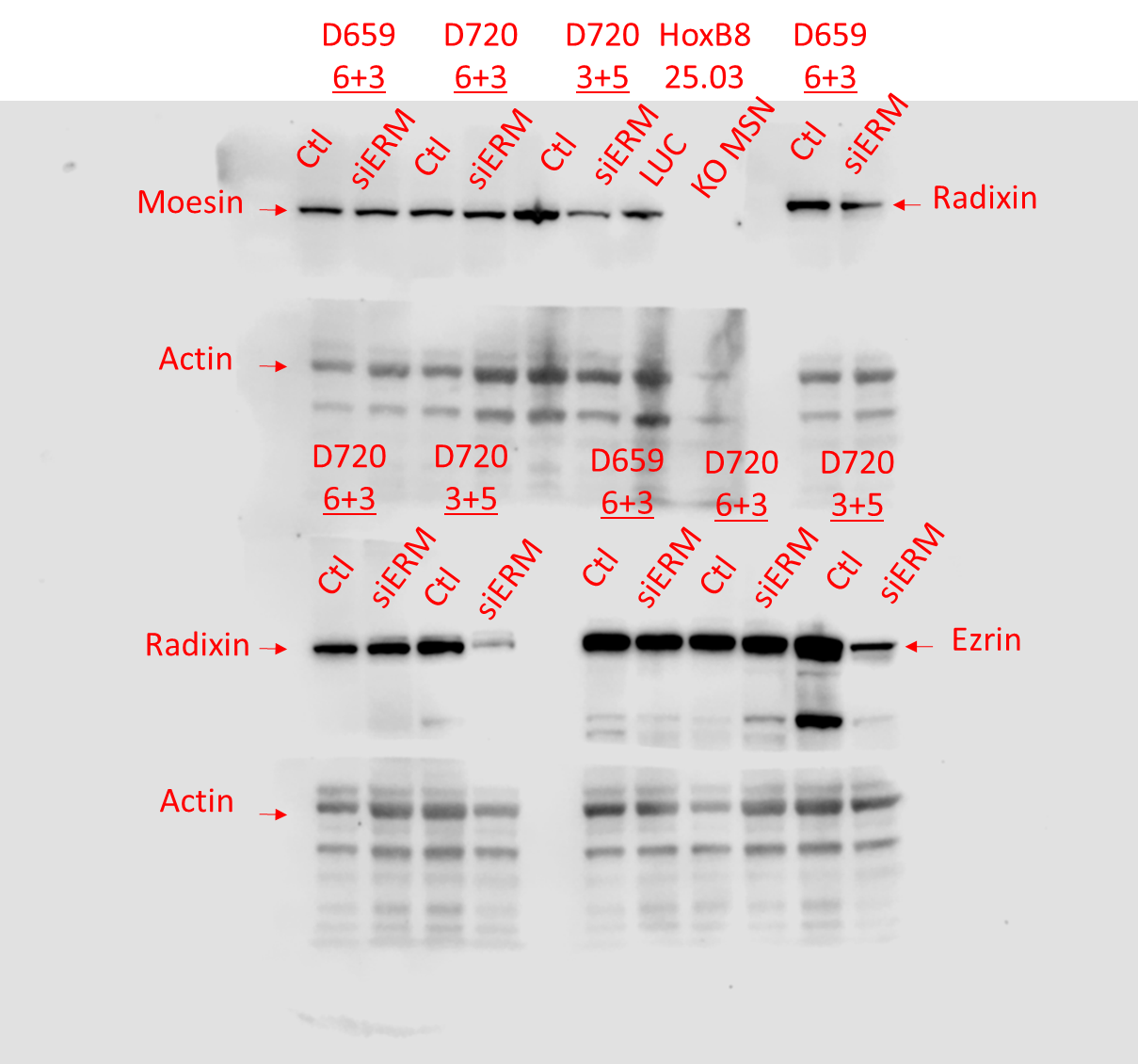

Supplement: Supplementary file 19 — Source data Fig. 1 [file 44318_2024_173_MOESM19_ESM.zip › Figure 1_ human macro_siMSN_Hox KO simples/1C WB/D720 original annotated high contrast.png]

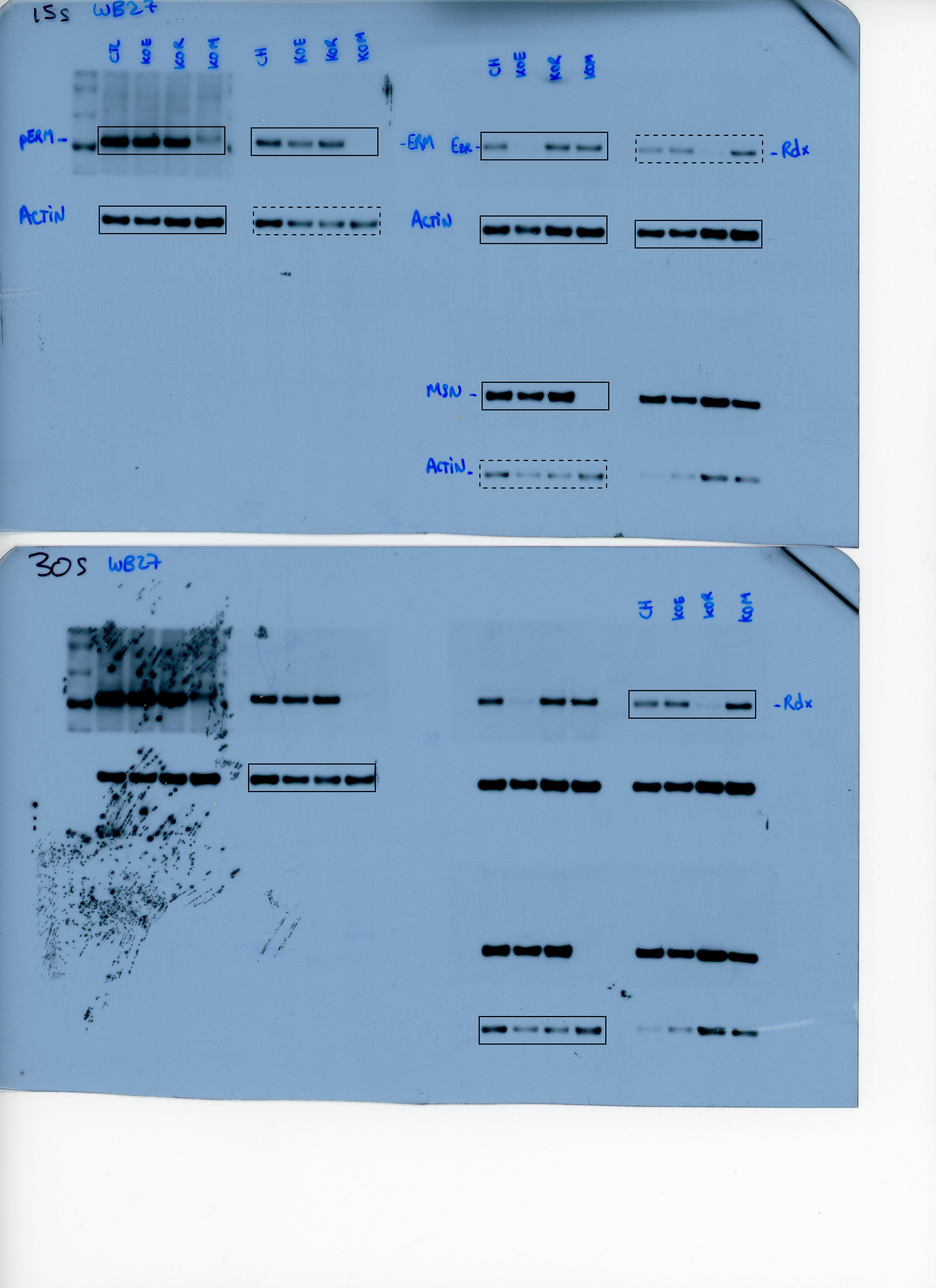

Supplement: Supplementary file 19 — Source data Fig. 1 [file 44318_2024_173_MOESM19_ESM.zip › Figure 1_ human macro_siMSN_Hox KO simples/1H Image Blot KO ERM simples/1H_simple ko_WB27_15s_30s_d.tif]

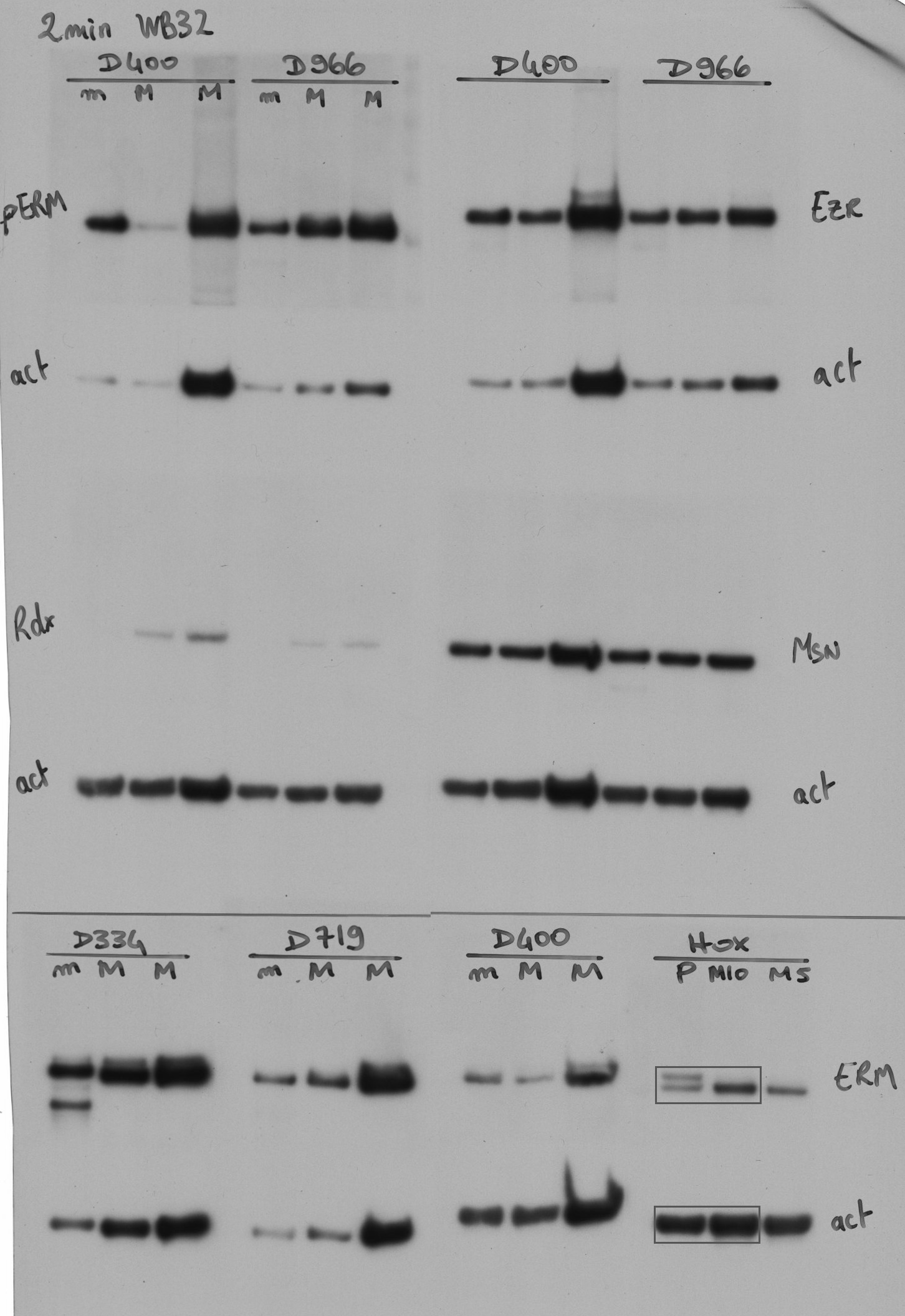

Supplement: Supplementary file 19 — Source data Fig. 1 [file 44318_2024_173_MOESM19_ESM.zip › Figure 1_ human macro_siMSN_Hox KO simples/1F Image Blot_hox ERM/WB32_2min_hox_ERM_actin.tif]

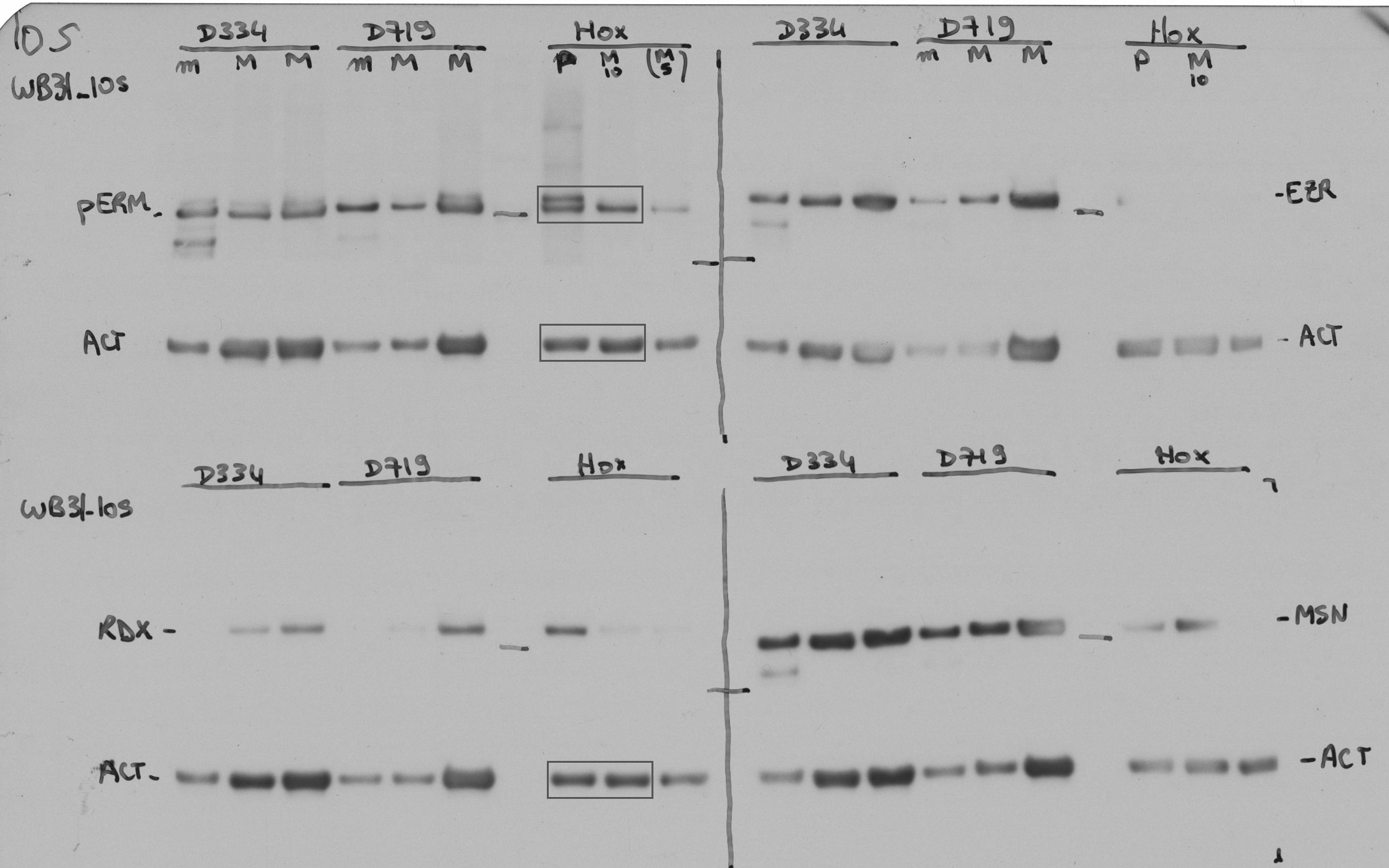

Supplement: Supplementary file 19 — Source data Fig. 1 [file 44318_2024_173_MOESM19_ESM.zip › Figure 1_ human macro_siMSN_Hox KO simples/1F Image Blot_hox ERM/WB31_10s_hox_pERM-actin_RDX-actin.tif]

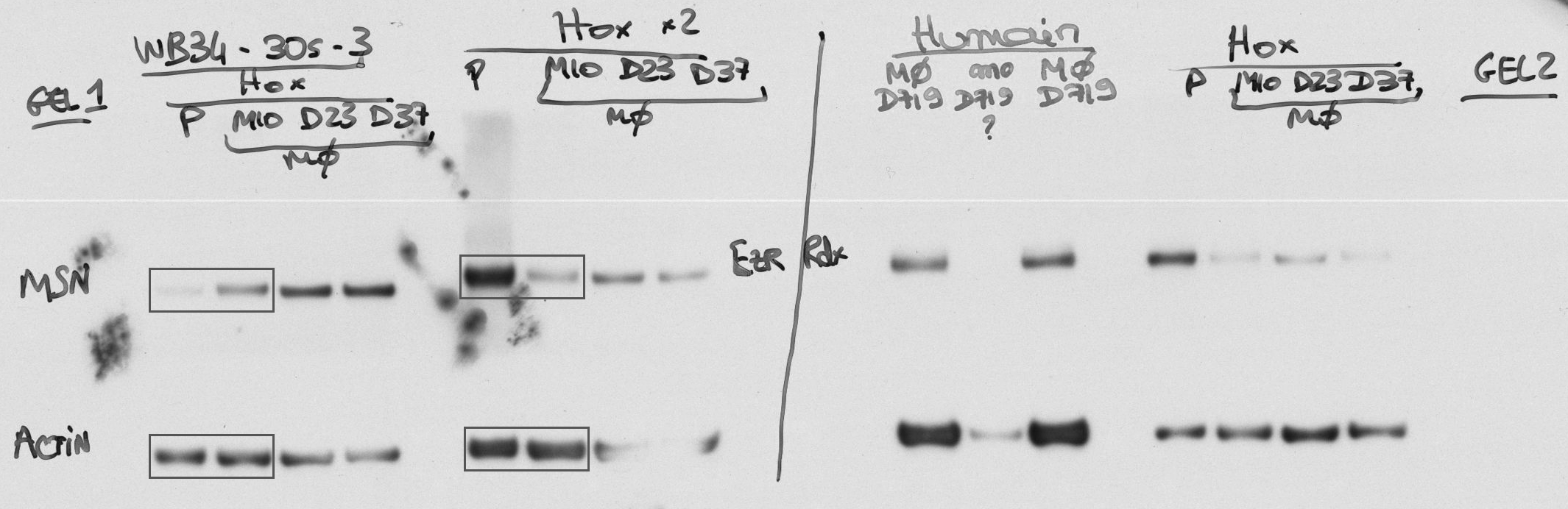

Supplement: Supplementary file 19 — Source data Fig. 1 [file 44318_2024_173_MOESM19_ESM.zip › Figure 1_ human macro_siMSN_Hox KO simples/1F Image Blot_hox ERM/WB34_30s_EZRIN_actin_MOESIN-actin.tif]

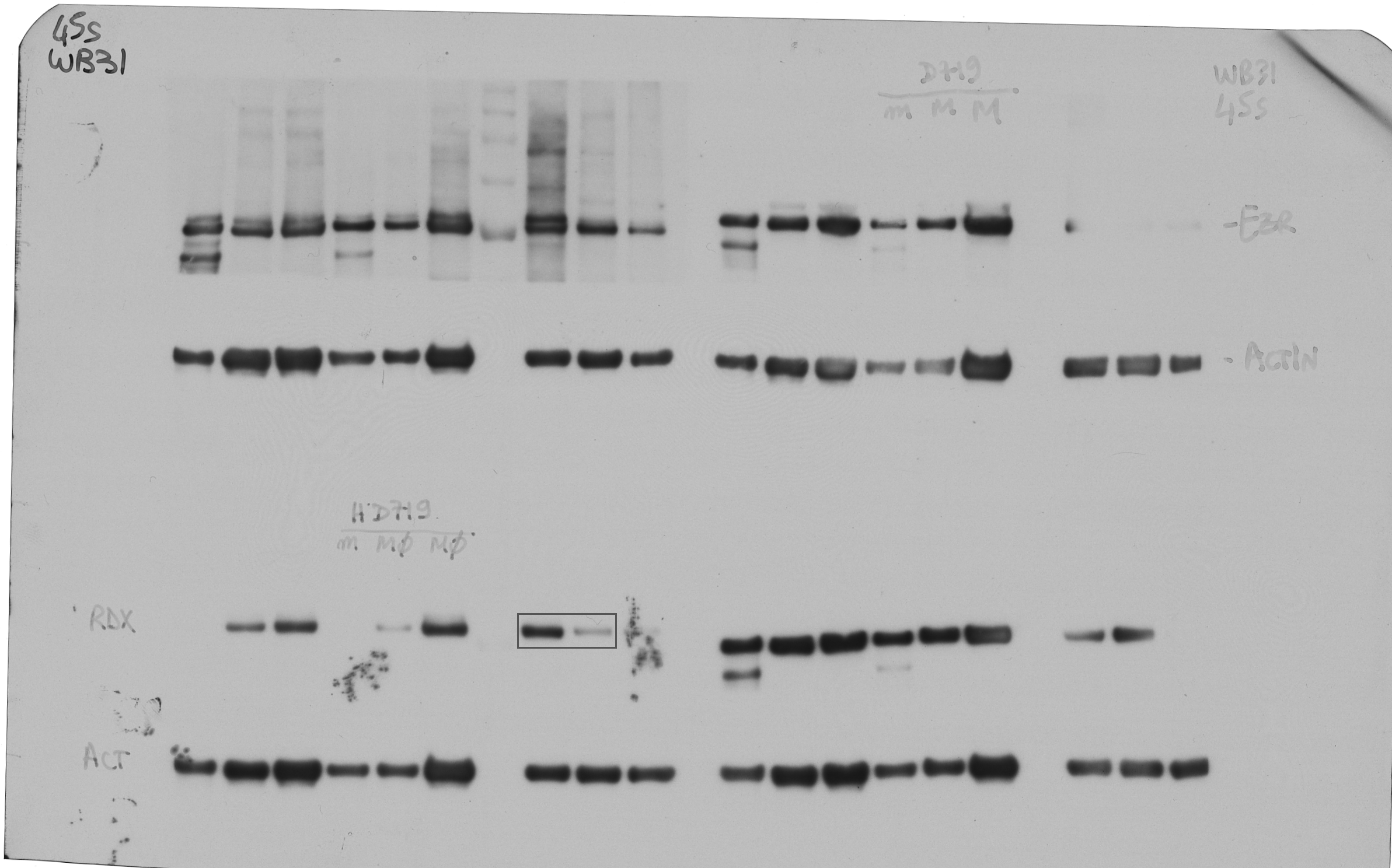

Supplement: Supplementary file 19 — Source data Fig. 1 [file 44318_2024_173_MOESM19_ESM.zip › Figure 1_ human macro_siMSN_Hox KO simples/1F Image Blot_hox ERM/WB31_45s_hox_RDX.tif]

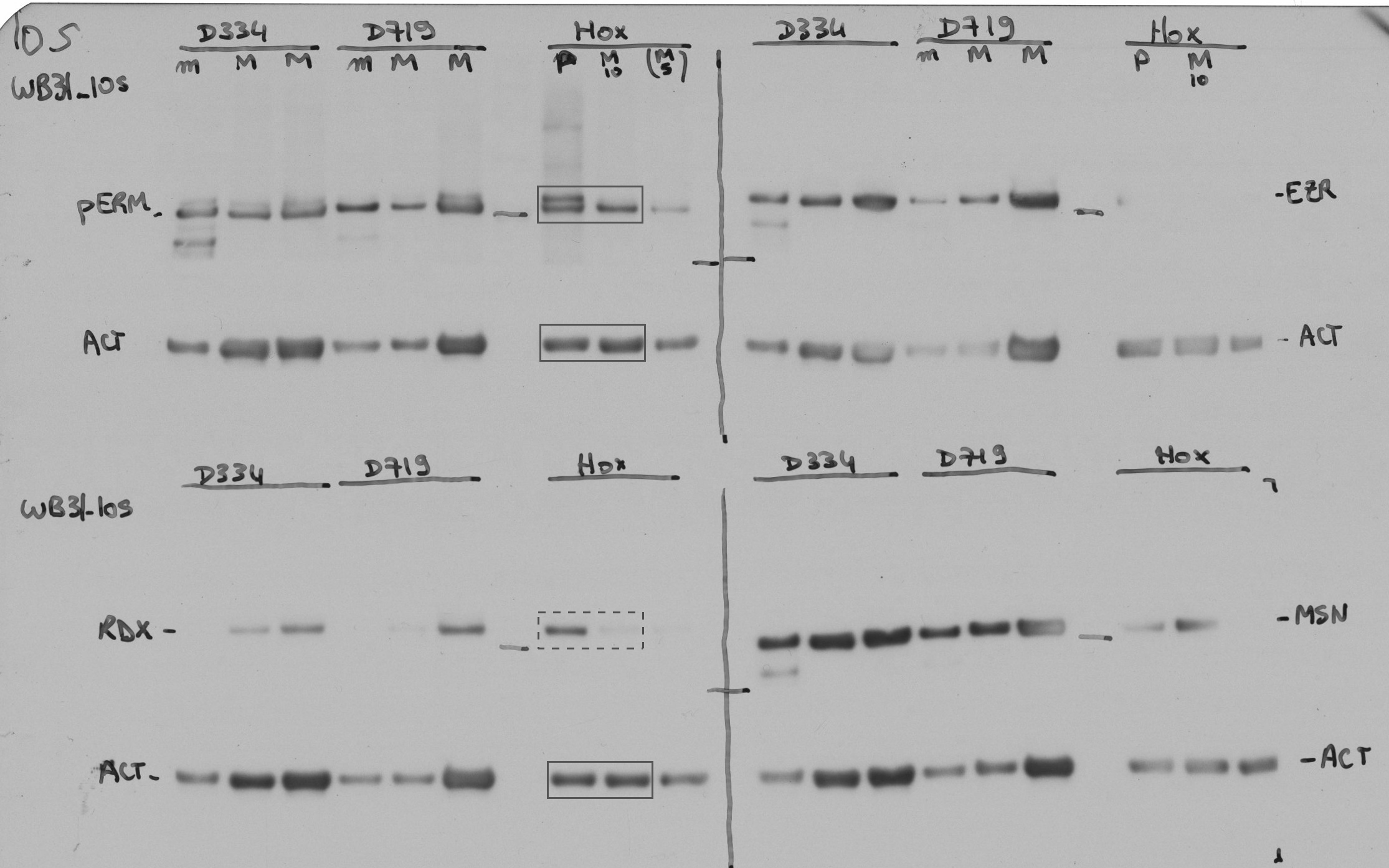

Supplement: Supplementary file 19 — Source data Fig. 1 [file 44318_2024_173_MOESM19_ESM.zip › Figure 1_ human macro_siMSN_Hox KO simples/1F Image Blot_hox ERM/WB31_10s_hox_pERM-actin_RDX-actin_dotted line.tif]

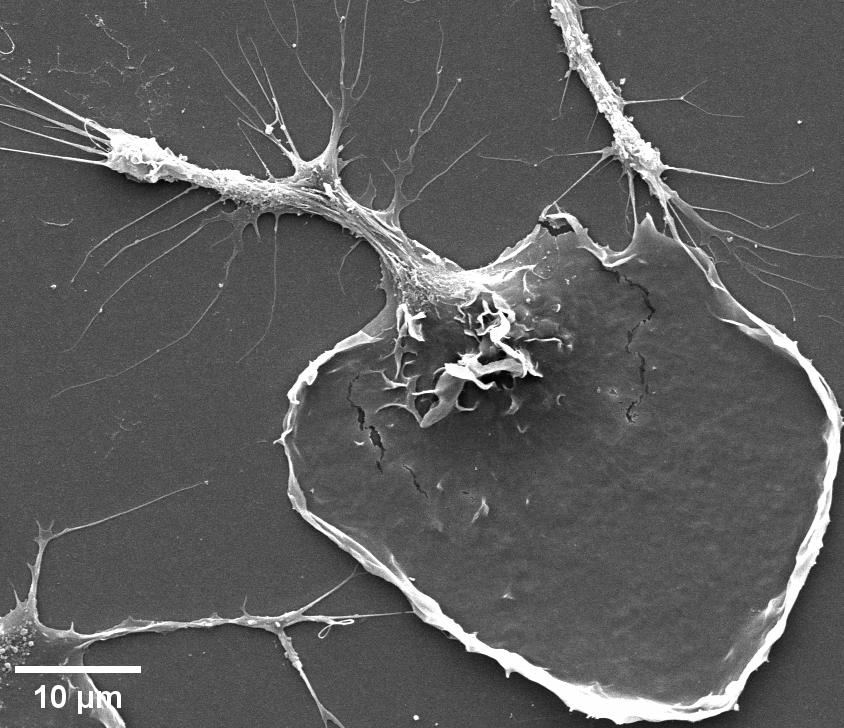

Supplement: Supplementary file 20 — Source data Fig. 2 [file 44318_2024_173_MOESM20_ESM.zip › Figure 2_TKO 2D Morpho/2C SEM macrophages/WT_macrophage.tif]

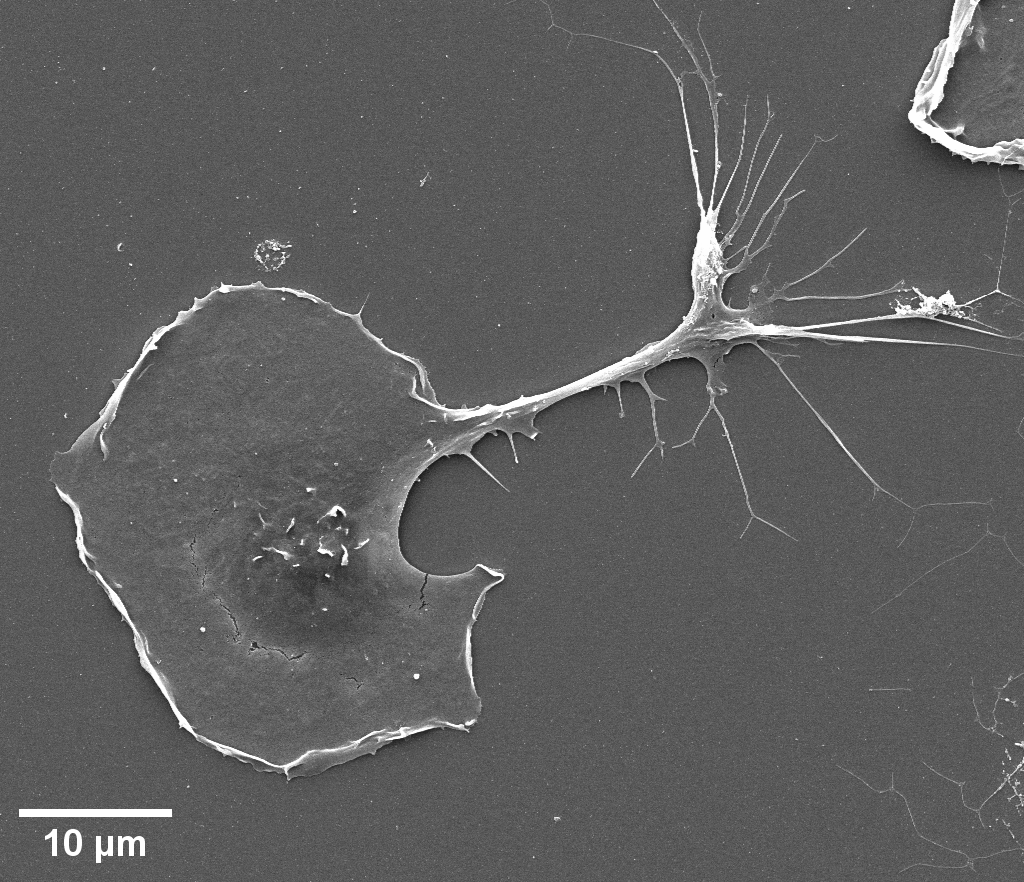

Supplement: Supplementary file 20 — Source data Fig. 2 [file 44318_2024_173_MOESM20_ESM.zip › Figure 2_TKO 2D Morpho/2C SEM macrophages/TKO#1_macrophage.tif]

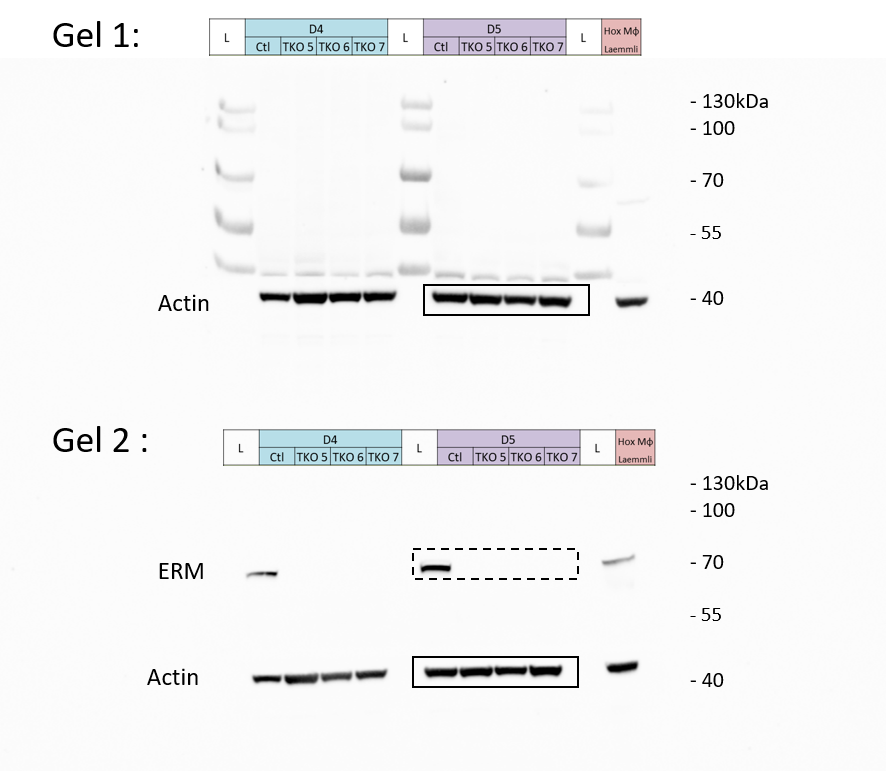

Supplement: Supplementary file 20 — Source data Fig. 2 [file 44318_2024_173_MOESM20_ESM.zip › Figure 2_TKO 2D Morpho/2A TKO WB/2A_1_WB22_revel1_10s_gel2-actin-ERM__gel1-actinforEzr.tif]

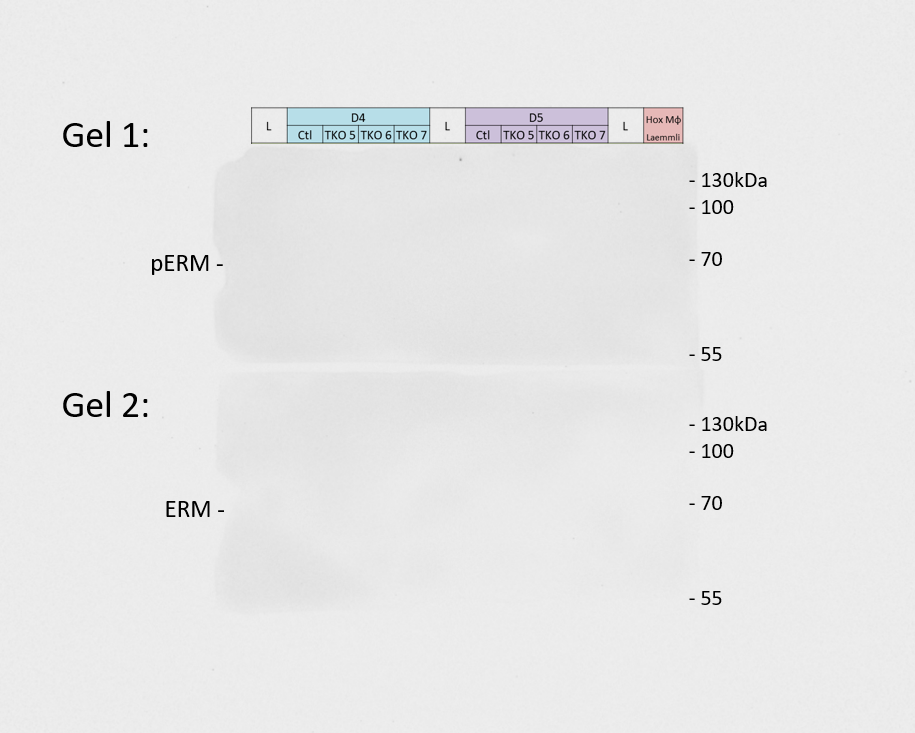

Supplement: Supplementary file 20 — Source data Fig. 2 [file 44318_2024_173_MOESM20_ESM.zip › Figure 2_TKO 2D Morpho/2A TKO WB/2A_3_WB22_revel_stripping_ERM-pERM.tif]

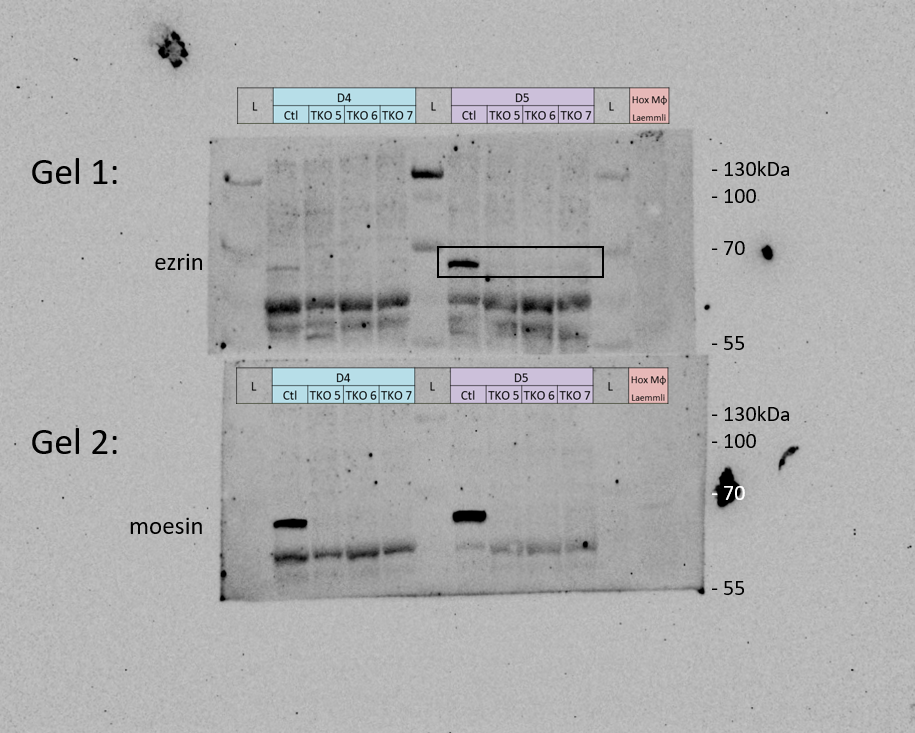

Supplement: Supplementary file 20 — Source data Fig. 2 [file 44318_2024_173_MOESM20_ESM.zip › Figure 2_TKO 2D Morpho/2A TKO WB/2A_4_WB22_revel2_2min_EZR_gel1.tif]

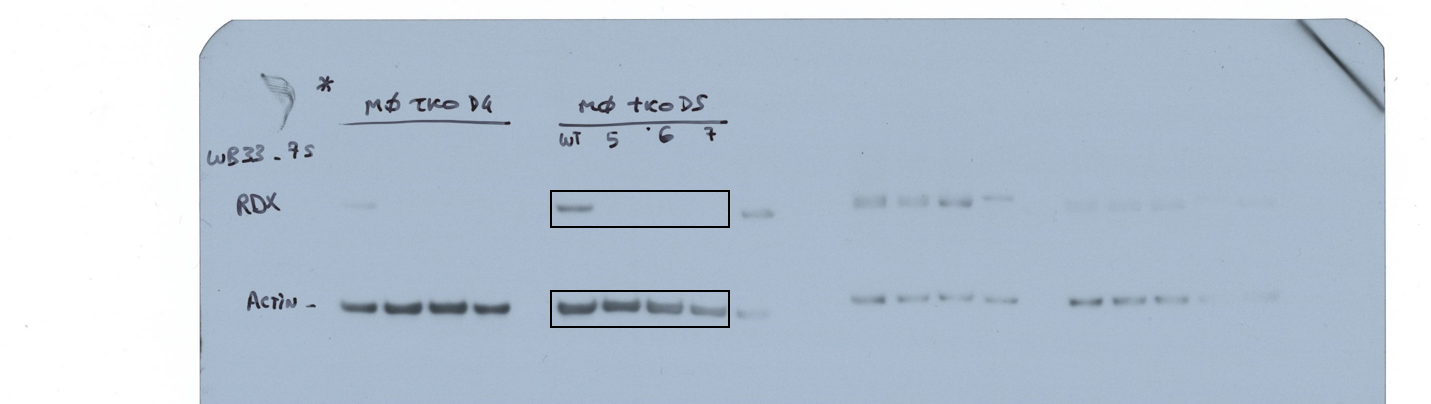

Supplement: Supplementary file 20 — Source data Fig. 2 [file 44318_2024_173_MOESM20_ESM.zip › Figure 2_TKO 2D Morpho/2A TKO WB/2A_8_WB33_7s_RDX.tif]

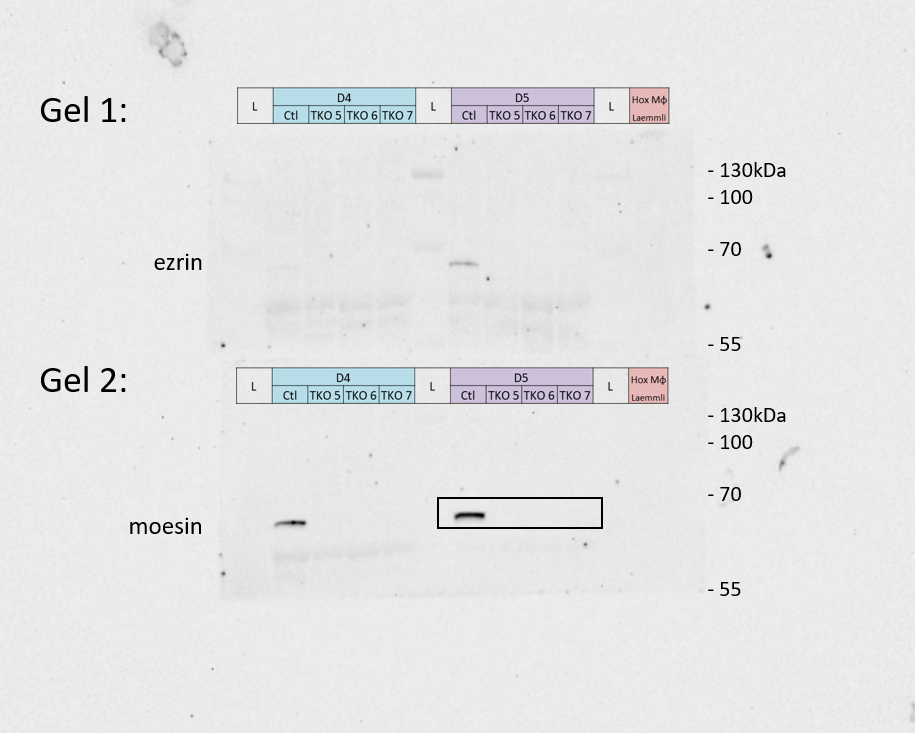

Supplement: Supplementary file 20 — Source data Fig. 2 [file 44318_2024_173_MOESM20_ESM.zip › Figure 2_TKO 2D Morpho/2A TKO WB/2A_5_WB22_revel2_45s_MSN_gel2.tif]

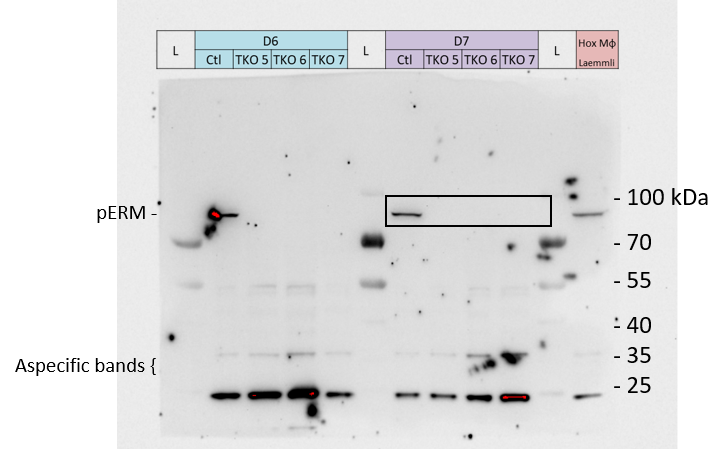

Supplement: Supplementary file 20 — Source data Fig. 2 [file 44318_2024_173_MOESM20_ESM.zip › Figure 2_TKO 2D Morpho/2A TKO WB/2A_6_WB23_gel1_1min_pERM_D6_D7.tif]

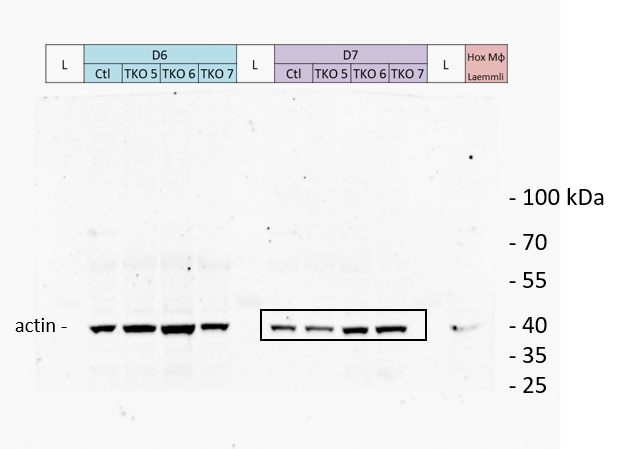

Supplement: Supplementary file 20 — Source data Fig. 2 [file 44318_2024_173_MOESM20_ESM.zip › Figure 2_TKO 2D Morpho/2A TKO WB/2A_7_WB23_gel1_20sec_Actin-pERM.tif]

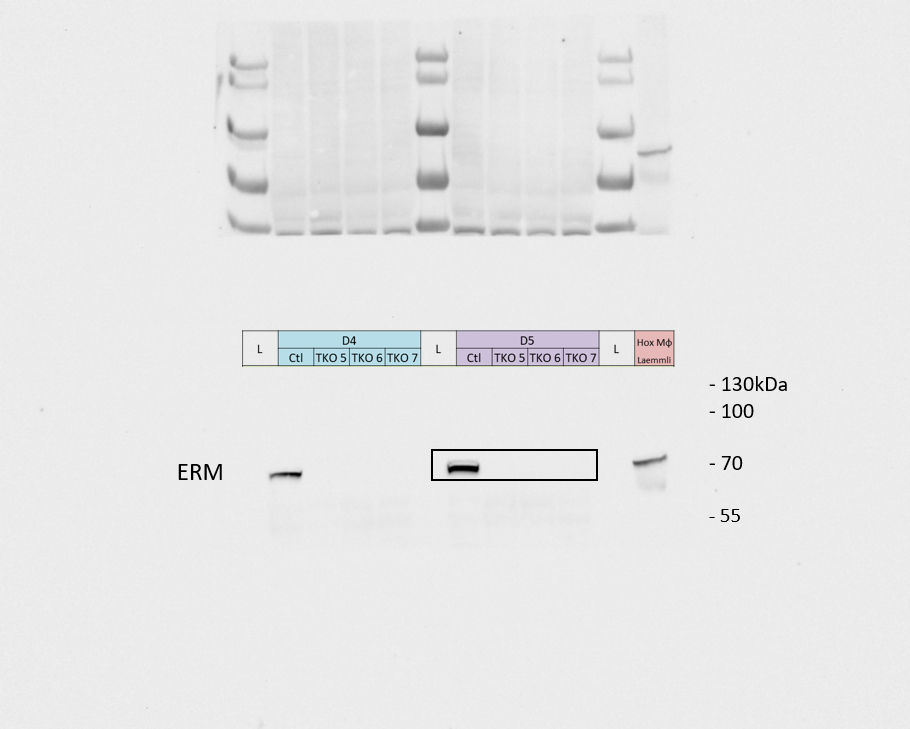

Supplement: Supplementary file 20 — Source data Fig. 2 [file 44318_2024_173_MOESM20_ESM.zip › Figure 2_TKO 2D Morpho/2A TKO WB/2A_2_WB22_revel1_30sec_gel2_erm_bas.tif]

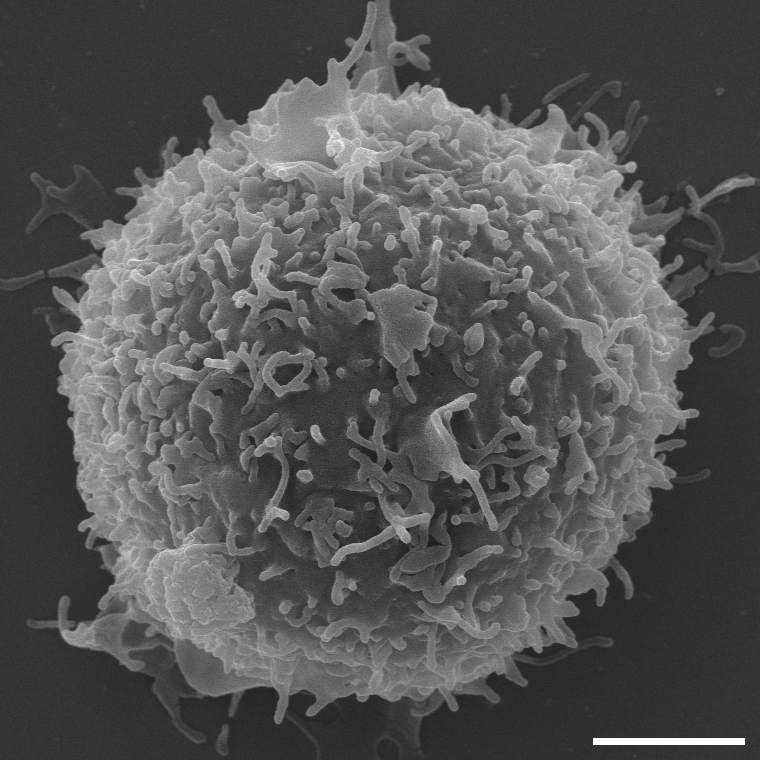

Supplement: Supplementary file 20 — Source data Fig. 2 [file 44318_2024_173_MOESM20_ESM.zip › Figure 2_TKO 2D Morpho/2B SEM progenitors/TKO#1_progenitor.tif]

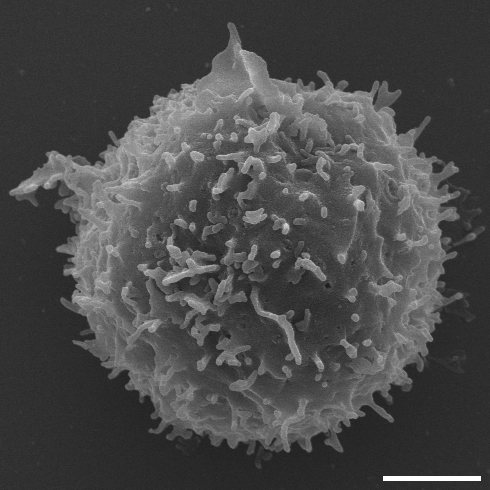

Supplement: Supplementary file 20 — Source data Fig. 2 [file 44318_2024_173_MOESM20_ESM.zip › Figure 2_TKO 2D Morpho/2B SEM progenitors/WT_progenitor.tif]

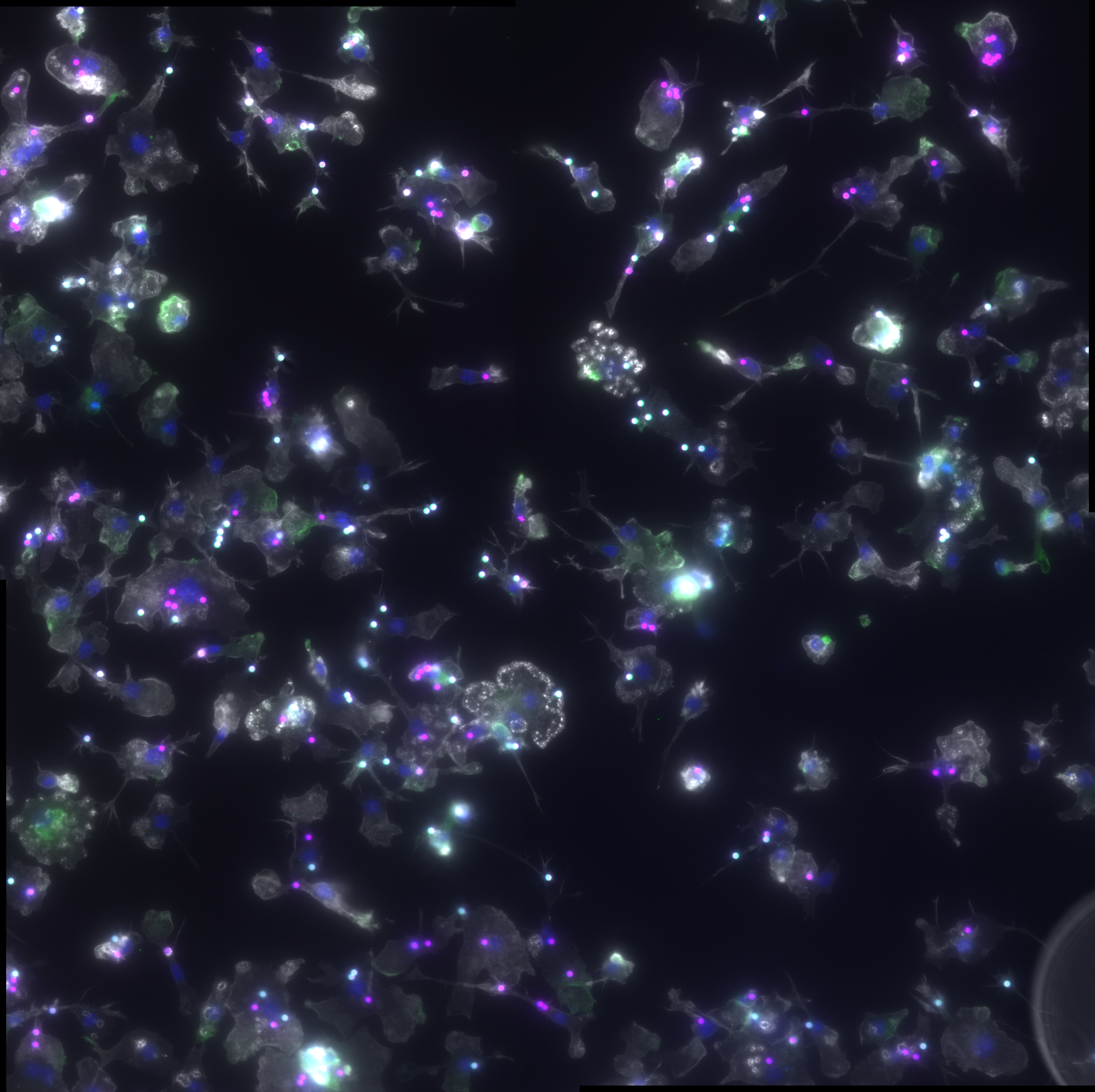

Supplement: Supplementary file 24 — EV Figure Source Data [file 44318_2024_173_MOESM24_ESM.zip › Figure EV3_Phagocytosis/EV3 A Images/tKO OVA 60'.tif]

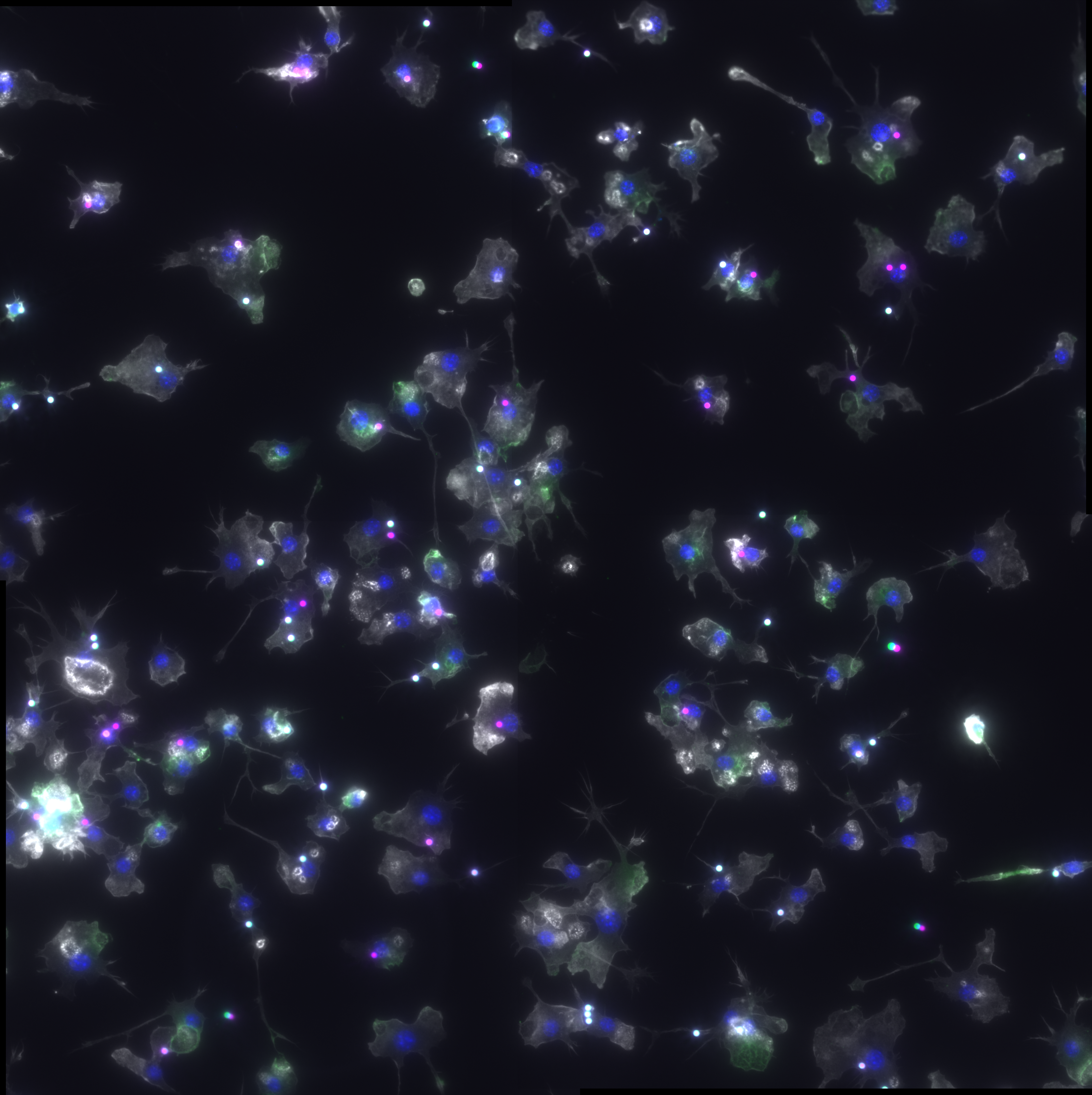

Supplement: Supplementary file 24 — EV Figure Source Data [file 44318_2024_173_MOESM24_ESM.zip › Figure EV3_Phagocytosis/EV3 A Images/WT OVA 60'.tif]
