# Supplementary material for: Ezrin, radixin, and moesin are dispensable for macrophage migration and cellular cortex mechanics
Source: EMBO J. 2024 Jul 18;43(21):4. doi: 10.1038/s44318-024-00173-7 (PMC11535515; doi:10.1038/s44318-024-00173-7)
Supplement: Supplementary file 25 — Expanded View Figures [file 44318_2024_173_MOESM25_ESM.pdf]

Expanded View Figures

Figure EV1. Localization of Ezrin, Radixin and Moesin proteins in human macrophages.

(**A–C**) Representative SIM images of HMDM co-transfected with ezrin-GFP (green) (**A**), radixin-GFP (green) (**B**) or moesin-GFP (green) (**C**) and Lifeact-mCherry (magenta) at the basal membrane, showing podosomes ($z = 0$ µm) and at 3 µm above the basal membrane, showing membrane ruffles (left panels). Scale bars: 10 µm, enlarged view: 1 µm. Intensity profiles along the dotted line from both enlarged view of left panels, crossing podosomes ($z = 0$ µm) and membrane ruffles ($z = 3$ µm) (right panels). Also see z-stack Movies EV1, 2 and 3. The fluorescence levels were adjusted in the same way in order to compare the intensity at the base of the cells to the upper planes. Note that ERM are mainly accumulated in the upper ruffles, compared to the basal plasma membrane and that only Ezrin slightly accumulate around podosome cores. (**D**) Enlarged view of ruffle dynamics from SIM images of HMDM co-transfected with ezrin-GFP (left panel), radixin-GFP (middle panel) or moesin-GFP (right panel) (green) and Lifeact-mCherry (magenta). Scale bars: 1 µm. ERM-GFP (green) or actin (magenta) intensity profiles along the dotted line are plotted below. Note that peripheral ruffles are enriched in F-actin, whereas ERM are present in both peripheral and central ruffles. Also see time-lapse Movies EV4, 5 and 6.

© The Author(s)

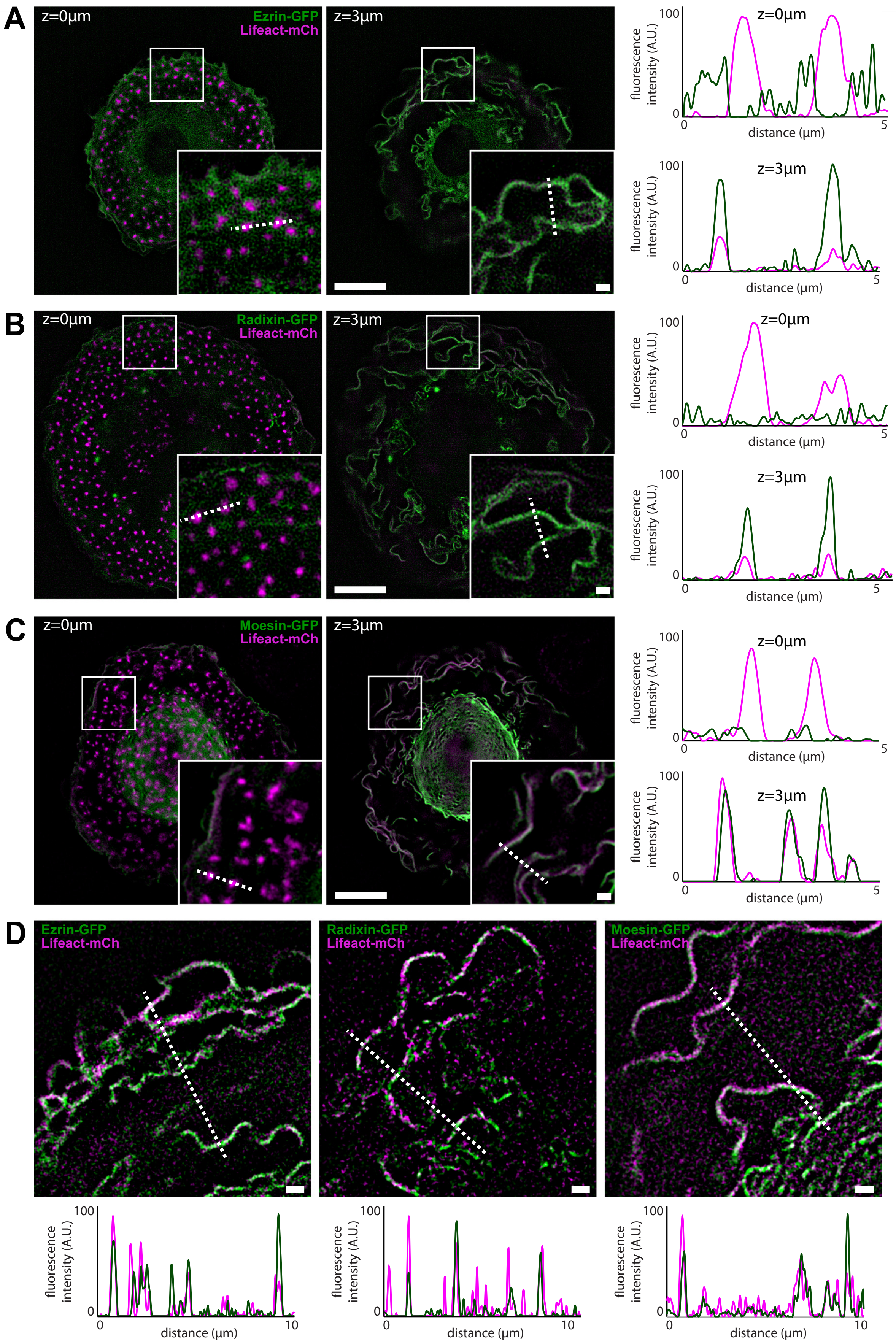
A
z=0µm
Ezrin-GFP
Lifeact-mCh
z=3µm
fluorescence intensity (A.U.)
100
0
distance (µm)
5
B
Radixin-GFP
Lifeact-mCh
C
Moesin-GFP
Lifeact-mCh
D
10

 © The Author(s)

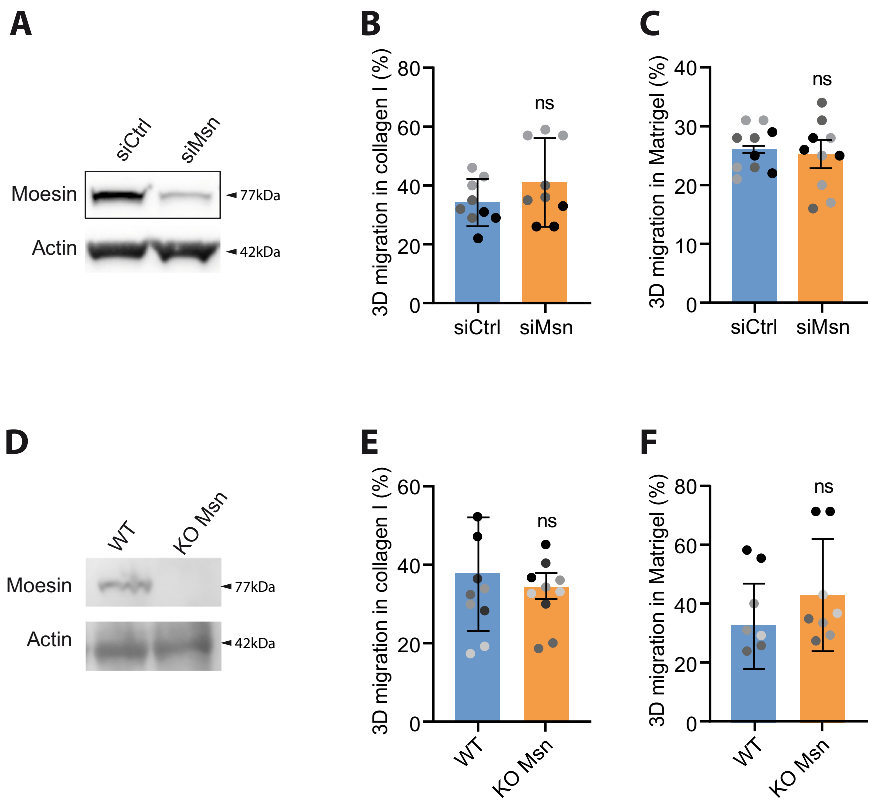

Figure EV2. Moesin siRNA and KO does not affect macrophage 3D migration.

(**A–C**) Depletion of Moesin in human macrophages by siRNA. (**A**) Moesin expression level of HMDM treated with siCtrl or siMoesin (siMSN) is representative of 3 independent donors. (**B**, **C**) Percentages of migration of siRNA-treated HMDM inside collagen I (**B**) and Matrigel (**C**) are represented as follows: the technical replicates (dot) of 3 independent experiments (highlighted by different gray colors) are represented. The mean (bar) and SD from the 3 independent experiments are shown. Statistical analysis was done on the mean per experiment using a paired two-tailed *t* test. (**D–F**) Moesin KO in mouse macrophages. (**D**) Moesin expression level in WT or Moesin KO mouse macrophages, differentiated in macrophage directly after KO induction to avoid compensations, is representative of 3 independent KO. (**E**, **F**) Percentages of migration inside collagen I (**E**) and Matrigel (**F**) are represented as follows: the technical replicates (dot) of 4 (collagen I) and 5 (Matrigel) independent experiments (highlighted by different gray colors) are represented. The mean (bar) and SD from the independent experiments are shown. Statistical analysis was done on the mean per experiment using a paired two-tailed t test.

© The Author(s)

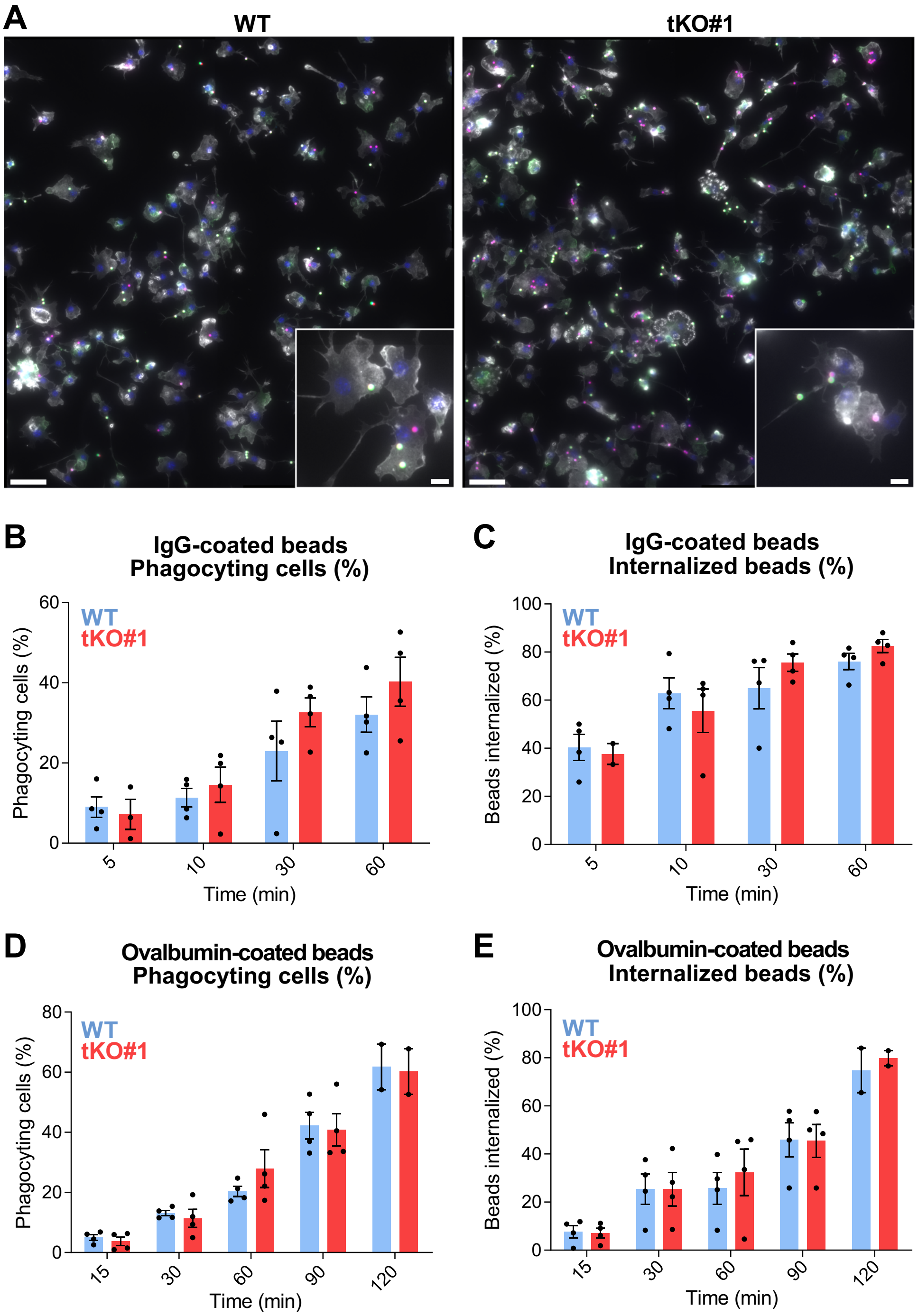

Figure EV3. Phagocytosis by WT and ERM-tKO macrophages.

HoxB8 macrophages were exposed to fluorescent IgG beads or OVA beads. Centrifugation was used to synchronize phagocytosis and cells were fixed at the indicated times (5 to 120 min). (**A**) Representative images of fluorescence microscopy of macrophages exposed to OVA beads for 60 min are shown. Beads that remained outside the cells were distinguished from ingested beads using anti-ovalbumin antibodies and TRITC-coupled secondary antibodies. Beads inside cells are magenta, beads outside cells are green, F-actin is shown in white and nuclei in blue. 3 × 3 tile images were stitched together with Zen software. Scale bars: 50 µm or 10 µm for zooms. (**B–E**) The percentages of phagocyting cells (**B**, **D**) and percentages of fully internalized beads (**C**, **E**) were quantified for both IgG beads and OVA beads. Results are expressed as mean +/− SD of at least 2600 cells/time point from 4 independent experiments and analyzed with two-way ANOVA followed by Boneferroni's comparison test, which revealed no significant differences. Source data are available online for this figure.

© The Author(s)

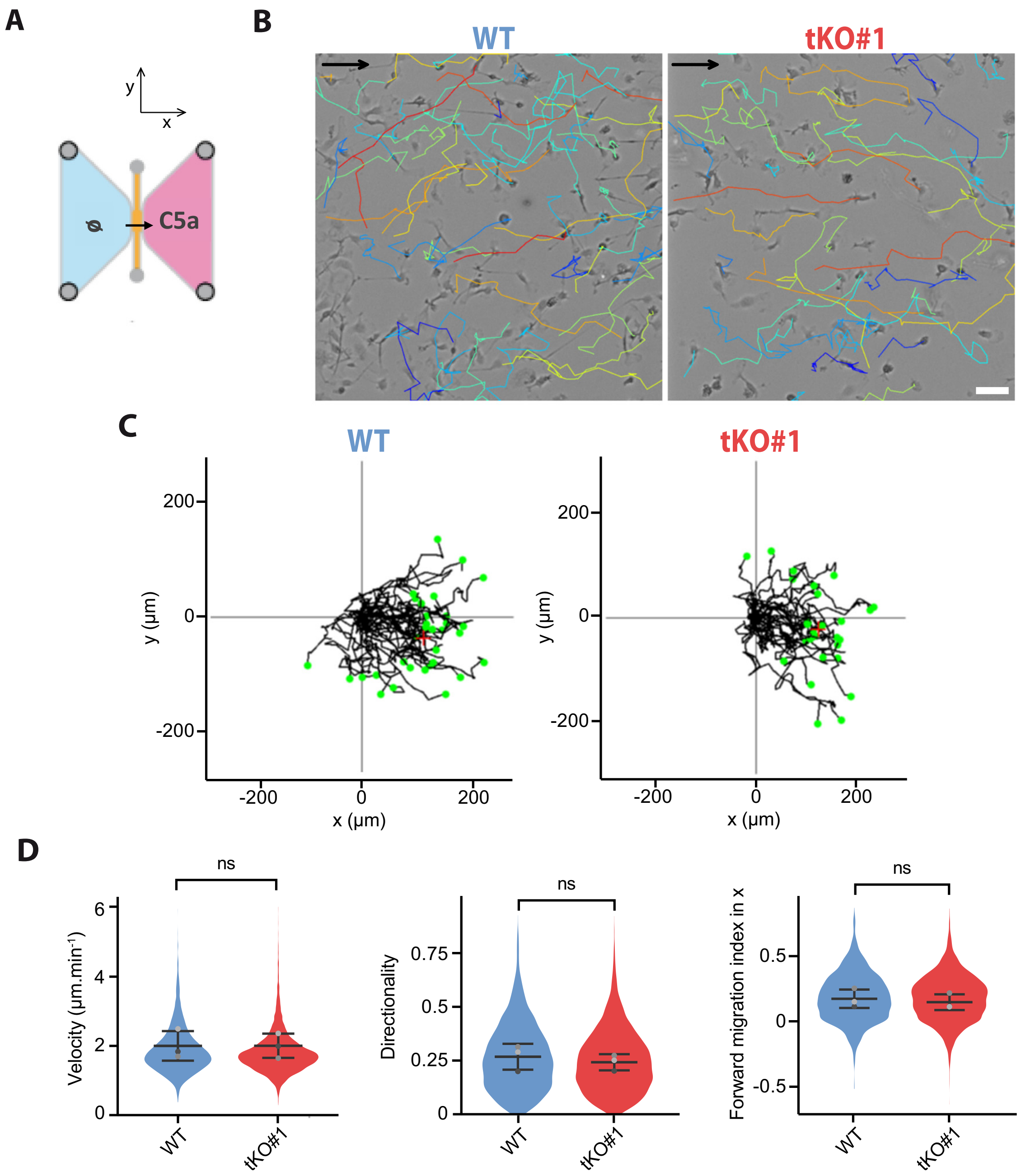

Figure EV4. 2D chemotaxis of WT and ERM-tKO#1 macrophages toward a C5a gradient.

(**A**) Schematic representation of 2D chemotaxis assay toward C5a. WT and ERM-tKO#1 cells migrating along a C5a gradient in the x axis. See also Movie EV9. (**B**) Snapshot of WT and ERM-tKO#1 macrophages migrating toward C5a gradient (on the right) with migratory tracks representing cell trajectories during 90 min. Tracks are color-coded according to their directionality. Scale bar: 50 µm. (**C**) Migratory tracks of WT and ERM-tKO#1 macrophages with origins set at (0,0). (**D**) Quantification of the median velocity, the directionality, and the forward migration index in the *x* axis (FMIx, used as a chemotaxis indicator) of each migratory track. The medians of 3 independent experiments are represented (gray points) and used for statistical analysis with a paired *t* test. Means and SD are shown.

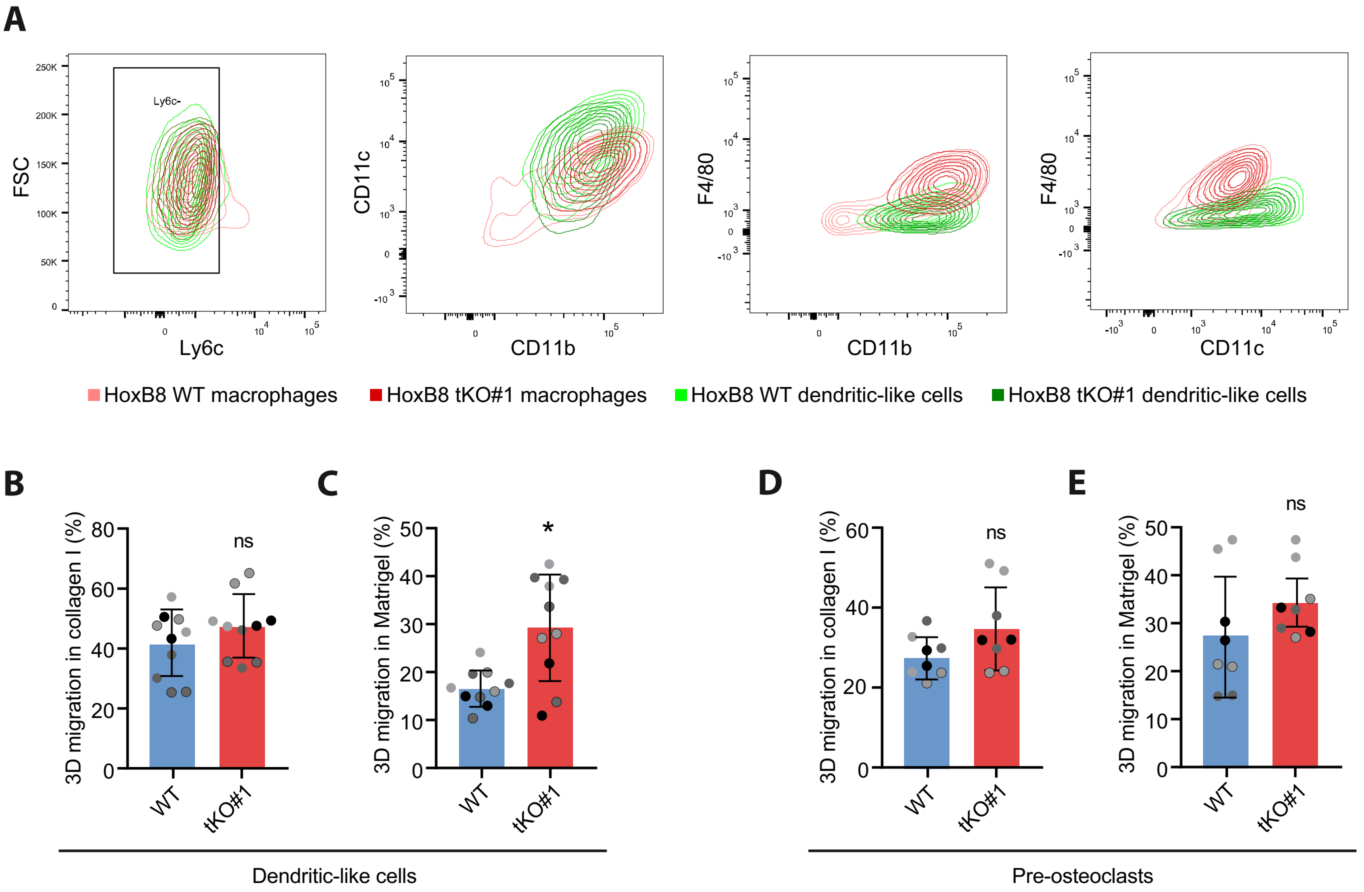

Figure EV5. ERM inhibit the 3D mesenchymal migration of dendritic-like cells.

(**A–C**) ERM-tKO affects the 3D migration through Matrigel of dendritic-like cells. (**A**) Differentiation of HoxB8 progenitors in dendritic-like cells. FACS analyses shows that HoxB8 progenitors differentiated with 40 ng/mL GM-CSF are Ly6C -, CD11b +, CD11c high, and F4/80 - dendritic-like cells (green), compared to M-CSF, which differentiates the same progenitors into Ly6C -, CD11b +, CD11c low and F4/80 + macrophages (red). (**B**, **C**) Percentages of migration of siRNA-treated HMDM inside collagen I (**B**) and Matrigel (**C**) are represented as follows: the technical replicates (dot) of 5 independent experiments (highlighted by different gray colors) are represented. The mean (bar) and SD from the 5 independent experiments are shown. Statistical analysis was done on the mean per experiment using a paired two-tailed *t* test. *$P < 0{,}05$. (**D**, **E**) ERM-tKO does not affect the 3D migration of pre-osteoclasts. Percentages of migration inside collagen I (**D**) and Matrigel (**E**) are represented as follows: the technical replicates (dot) of 4 independent experiments (highlighted by different gray colors) are represented. The mean (bar) and SD from the independent experiments are shown. Statistical analysis was done on the mean per experiment using a paired two-tailed *t* test.

 © The Author(s)
